# Identification and characterization of novel *SUMO* genes in bread wheat

**DOI:** 10.7717/peerj.20432

**Published:** 2025-11-28

**Authors:** Eid I. Ibrahim, Kotb A. Attia, Abdelhalim Ghazy, Itoh Kimiko, Abdullah Al-Doss

**Affiliations:** 1 Plant Production Department, College of Food and Agriculture Sciences, King Saud University, Riyadh, Saudi Arabia; 2 Center of Excellence in Biotechnology Research, King Saud University, Riyadh, Saudi Arabia; 3 Institute of Science and Technology, Niigata University, Niigata, Japan

**Keywords:** SUMO genes, Wheat, Bioinformatics, Gene/Protein expression

## Abstract

The small-ubiquitin-like-modifier (SUMO), composed of approximately 100 amino acid residues, regulates the cellular activity of several proteins by posttranslational modification. Almost all plant species express a family of SUMO isoforms. Nevertheless, the *SUMO* genes in wheat (*TaSUMOs*) have not undergone complete characterization, and the roles of *TaSUMOs* remain unidentified. The study identified four new *SUMO* genes in wheat, named *TaSUMO4-7*, in addition to the previously known *TaSUMO1-3*. These genes are part of the conserved SUMO family, as indicated by phylogenetic analysis. The genes contain the characteristic SUMO-acceptor site motif and the essential C-terminal diglycine motif for processing. Expression analysis showed that *TaSUMO4-7* genes are expressed in various wheat tissues. Bioinformatics analysis predicted the biochemical properties and structures of the proteins, which were found to localize in the cytoplasm and nucleus. The study confirms that the new *TaSUMO4-7* genes are functional members of the wheat SUMO family and lays the groundwork for further research into their specific roles.

## Introduction

Among the various posttranslational modifications (PTMs) crucial for cellular function in eukaryotes, SUMOylation stands out for its similarity and significance alongside the widely studied ubiquitination pathway. However, diverse PTMs, including phosphorylation, lipidation, glycosylation, acetylation, and ubiquitination, all contribute to regulating various cellular processes (Kerscher, Felberbaum & Hochstrasser, 2006; Clague, Coulson & Urbé, 2012; Isono & Nagel, 2014; Wang, Peterson & Loring, 2014). In contrast to ubiquitination, which mainly aims to degrade substrates, SUMOylation controls substrate functions primarily by changing protein protein interactions, intracellular localization or other forms of PTMs (Zhao, 2007). SUMOylation is a vital controlling tool in eukaryotic cells that influences diverse processes, such as enzyme stability and activity, subnuclear localization of proteins, cell cycle regulation, and DNA repair (Miura, Jin & Hasegawa, 2007a). SUMOylation is achieved through a multistep enzymatic pathway involving three key players: E1 (a small-ubiquitin-like-modifier (SUMO)-activating-enzyme), E2 (a SUMO-conjugating-enzyme), and E3 (a SUMO-ligating-enzyme) (Miura, Jin & Hasegawa, 2007a).

SUMOylation refers to the modification of a substrate using a small-ubiquitin-like modifier. SUMO belongs to the category of low-molecular-weight proteins, which typically contain between 100 and 130 amino acids (Kim, Baek & Chung, 2002). In 1999, SUMO was first discovered in tomato plants as an interaction partner with the plant pathogenic fungus *Trichoderma viride’*s ethylene-inducing-xylanase (EIX) (Hanania et al., 1999). Ubiquitous across eukaryotic cells, the SUMO protein is known as a crucial controlling mechanism that regulates various cellular pathways through targeted protein modification (Morrell & Sadanandom, 2019; Le Roux et al., 2019). Unlike worms, fruit flies, and yeasts, plants express numerous SUMO isoforms that may indicate SUMO crosstalk and/or substrate specificity (Flotho & Melchior, 2013; Van den Burg et al., 2010). Seven *SUMO*-genes have been discovered in *Oryza sativa* (Ikarashi et al., 2012; Rosa et al., 2018; Ibrahim et al., 2021; Teramura et al., 2021), and three *SUMO*-genes have been recognized in wheat (Wang et al., 2018); while, in the *Arabidopsis thaliana* genome, eight *SUMO*-genes have been reported (Kurepa et al., 2003; Novatchkova et al., 2004; Hay, 2005).

The SUMO-precursor is shacked in the C terminal GG motif *via* a SUMO-specific-protease and a subsequent pathway involving E1 (SAEs), E2 (SCEs), and E3 (SLEs), which tie mature SUMO to the certain protein by covalently binding to lysine (K) residues (Bernier-Villamor et al., 2002; Lois & Lima, 2005; Yunus & Lima, 2006; Miura et al., 2007b; Yaeno & Iba, 2008; Nayak & Müller, 2014). The attachment of SUMO to a certain protein (substrate) is not permanent but rather a dynamic process controlled by SUMO proteases. These enzymes are believed to be crucial regulators of SUMOylation activity (Le Roux, 2016). Most SUMO-targeted proteins possess a specific recognition motif known as the consensus pattern, typically represented as ΨKXE/D. This pattern includes a hydrophobic residue (Ψ) followed by a lysine (K), then any amino acid (X), followed by either glutamic acid (E) or aspartic acid (D) (Lois, Lima & Chua, 2003; Rosa et al., 2018; Morrell & Sadanandom, 2019).

*SUMO* genes are believed to be vital for regulating a range of environmental interactions. It has been shown to control a variety of plant biological mechanisms, like abiotic (Van den Burg et al., 2010; Augustine & Vierstra, 2018; Han et al., 2022) and biotic (Flick & Kaiser, 2009) stresses tolerance, plant development (Castro et al., 2018), root growth (Zhang, Qi & Yang, 2010) and plant reproduction (Augustine et al., 2016). While the importance of SUMOylation and its related genes is well established, significant gaps remain in our understanding of *SUMO* genes in bread wheat. These gaps encompass their specific functions, evolutionary connections to other crops, and potential involvement in managing diverse environmental stresses. Given the crucial role of SUMOs in agriculturally relevant traits, elucidating the specific functions of SUMOs in wheat has immense potential for enhancing both grain quality and yield.

While the study by Wang et al. (2018) successfully identified three *TaSUMO* genes (*TaSUMO1–3*) in wheat through homology-based searches using Arabidopsis and rice sequences, their approach did not fully capture the diversity of the *SUMO* gene family in the wheat genome. Our study expands upon their findings by employing more stringent bioinformatic criteria (*E*-value <1.0^*e*−5^, % identity ≥ 50) and additional validation steps, including Pfam and InterPro domain analysis, to ensure the accuracy of gene identification. Crucially, we combined computational predictions with experimental validation, using PCR amplification, sequencing, and tissue-specific expression profiling to confirm the existence of four new *TaSUMO* genes (*TaSUMO4–7*). Furthermore, we conducted functional characterization through subcellular localization assays (*via* fluorescent protein tagging and confocal microscopy) and interaction studies with the SUMO-conjugation enzyme *SCE1a*, providing mechanistic insights that were absent in the earlier work. This study lays the groundwork for future in-depth investigations of the *TaSUMO* gene family by utilizing powerful bioinformatic tools and insights.

## Materials & Methods

### Identification of *SUMO* genes in wheat

Three wheat *SUMO*-genes have been reported (Wang et al., 2018) specifically *TaSUMO1* (TraesCS3A02G424000.1, TraesCS3B02G460000.1, TraesCS3D02G419300.1), *TaSUMO2* (TraesCS3B02G459900.1, TraesCS3D02G419200.1), and *TaSUMO3* (TraesCS3A02G366700.1, TraesCS3D02G359600.1). We obtained their sequences from the Ensembl Plants (http://plants.ensembl.org/). To identify new *SUMO* genes in *Triticum aestivum* L. (*TaSUMOs*), *SUMO* sequences in *Arabidopsis thaliana* (*AtSUMOs*) were obtained from TAIR (http://www.arabidopsis.org, accessed on 16 January 2022) and rice *SUMO* sequences (*OsSUMOs*) derived from the Rice Annotation Project (https://rice.uga.edu/, accessed on 16 January 2022) were used. These sequences served as queries for exploring the wheat database within the Ensembl Plants datasets (http://plants.ensembl.org/Triticum_aestivum/Info/Index, accessed on 17 January 2022). To ensure the accuracy of subsequent analyses, stringent filtering criteria were applied (E-val < 1.0^*e*−5^, %ID ≥ 50). This involved removing redundant genes and selecting the longest transcript representative of each gene family member. Further validation of the identified TaSUMO family members was conducted using the InterPro website (https://www.ebi.ac.uk/interpro/, accessed on 19 January 2022) by submitting the predicted protein sequences.

### Plant material growth and DNA extraction

Seeds of *Triticum aestivum* L. cv. VEERY (Chilean variety) were germinated in a controlled environment chamber under the following conditions: 23 °C ± 2 °C throughout the day, 16 °C ± 2 °C at night, 70% relative humidity, 16 h of light and 8 h of darkness (long-day conditions), and a light intensity of approximately 60 µmol m^−^^2^ s^−^^1^ at the Plant Production Department, College of Food and Agriculture Sciences, King Saud University, Riyadh, Saudi Arabia. The cultivar grains were obtained from the International Maize and Wheat Improvement Center (CIMMYT). The molecular experiments were conducted at the Biotechnology Laboratory within the Plant Production Department, KSU. Total DNA was extracted from young wheat leaves *via* the Wizard^®^ Genomic DNA Purification Kit manufactured by Promega (Madison, WI, USA).

### Candidate gene amplification and sequencing of putative *TaSUMOs*

The putative *TaSUMO4-7* sequences were amplified by the definite primers presented in Table S1. These primers were designed based on the genomic data of the *SUMO*-region in the *Triticum aestivum* genome database. The PCR amplification involved the addition of 10 µL of GoTaq^®^ Green Master Mix (1 ×) (Promega Corporation, Madison, WI, USA), two µL of mixed primers, six µL of water, and two µL of DNA. The cycling procedure was 95 °C for 5 min (initial denaturation), followed by 35 cycles of 95 °C for 30 s (denaturation), 52−64 °C for 30 s (annealing), 72 °C for 1 min (extension), and a final extension at 72 °C for 7 min. Amplified DNA (10 µL) was visualized on a 3% agarose gel. The expected bands were purified with a QIAquick^®^ PCR Purification Kit and sequenced by Macrogen^®^ (Seoul, South Korea). The coding regions were analysed for SUMO family characteristics.

### Bioinformatic characterization of *TaSUMOs*

Characterization of the *TaSUMO* gene family included features such as amino acid count, chromosomal location, gene ID, length of coding sequences (CDSs), and exon number extracted from the Ensembl Plants database. The biochemical characteristics of the SUMO proteins were analysed using the ExPASy ProtParam server tool (https://web.expasy.org/protparam/, accessed on 20 September 2025) (Gasteiger et al., 2003). This included calculating various parameters of the primary structure, like the amino acid composition, molecular weight (MW), and theoretical isoelectric point (T-pi). The grand average of hydropathicity (GRAVY) was calculated using the method described by Kyte & Doolittle (1982), while the instability index (Ii) was calculated using the method described by Guruprasad, Reddy & Pandit (1990). This latter analysis considers the frequency of specific dipeptides as an indicator of protein stability, with proteins having an Ii less than 40 considered stable and those above 40 considered unstable. Finally, the aliphatic index (Ai) was also calculated using the formula proposed by Ikai (1980).

The Protein Homology/analogY Recognition Engine V 2.0, Phyre2 web portal (http://www.sbg.bio.ic.ac.uk/phyre2/html/page.cgi?id=index/, accessed on 21 January 2022) (Kelley et al., 2015) was employed to computationally predict the secondary and three-dimensional (3D) structures of the proteins. Computational prediction of SUMO-binding sites within the TaSUMO genes was performed using the SUMOplot™ Analysis Program (https://www.abcepta.com/sumoplot/, accessed on 17 January 2022). Additionally, the pairwise sequence identity among the TaSUMO protein sequences was detected *via* Vector NTI^®^ Software (Invitrogen, Waltham, MA, USA).

### TaSUMO sequence comparison

Phylogenetic analysis incorporated diverse data sets, including the three identified TaSUMO protein sequences from Wang et al. (2018), eight AtSUMO sequences reported by Kurepa et al. (2003), Novatchkova et al. (2004), and Hay (2005), and seven OsSUMO sequences described by Ikarashi et al. (2012), Rosa et al. (2018), Ibrahim et al. (2021), Teramura et al. (2021), and Ibrahim (2022). SUMO protein sequences from humans, yeast, and other species were got from the National Center for Biotechnology Information (NCBI) and the UniProt database. Multiple sequence alignments were performed through the UniProt database website (https://www.uniprot.org/align/, accessed on 17 January 2022) using the ClustalO function. The SUMO protein sequences were queried against the InterPro website (Finn et al., 2007; Larkin et al., 2007) to identify conserved domains characteristic of the SUMO family. A phylogenetic tree was constructed using the neighbor−joining method (Saitou & Nei, 1987) with the Poisson correction model and 1,000 bootstrap replicates (Felsenstein, 1985) implemented in MEGA X software (Kumar et al., 2018). This method was subsequently applied to generate a specific phylogenetic tree of TaSUMO protein sequences.

### RNA extraction and RT–PCR

To determine the transcription levels of the *TaSUMO4-7* genes, various parts of the plants were collected, frozen in liquid nitrogen, and ground. RNA was subsequently extracted with a commercial kit (RNeasy^®^ Plant Mini Kit, Qiagen, Hilden, Germany). The extracted RNA was used to create first-strand cDNA copies (SuperScript II, Invitrogen, Waltham, MA, USA). RT−PCR was subsequently performed by a specific enzyme (KOD Dash DNA polymerase, Toyobo, Osaka, Japan) and a thermal cycler (Veriti 96-well, Applied Biosystems, Waltham, MA, USA). Gene-specific primers for the putative *TaSUMO* genes (Table S1) were used to amplify their transcripts, and wheat actin served as an internal control for normalization.

### Construction of expression vectors harboring *TaSUMOs*

We constructed expression vectors containing *TaSUMO* components using the *pUC119* vector (Vieira & Messing, 1989) and protocols from Ikarashi et al. (2012). These vectors expressed fluorescent protein tags, either red (DsRFP) or green (GFP), under the regulation of the cauliflower mosaic virus 35S (CaMV35S) promoter (Fig. S1). All primers used are presented in Table S1. The new genes were introduced into pDsRFP *via* an In-Fusion HD Cloning Kit (Takara Bio) to create vectors named *pDsRFP:TaSUMO4-7*, which were used to study the localization and expression of the new TaSUMO proteins. Additionally, *pDsRFP:TaSUMO4-7*Δ*GG* vectors with deleted GG motifs were constructed to understand their role in protein localization. To study the coexpression and localization with TaSUMO4-7, the *Arabidopsis thaliana* SUMO-conjugation-enzyme (SCE1a) was inserted into the *pGFP:SCE1a* vector. Furthermore, cell organelle markers (peroxisome, mitochondria, plastid, cis-Golgi) were fused with GFP in vectors (*pPTS2:GFP, pmt:GFP, pWxTP:GFP, pGFP:SYP31*) and cotransfected with *DsRFP:TaSUMO4-7* in onion cells to determine whether TaSUMO4-7 proteins localize to specific organelles (Fig. S1).

### TaSUMOs, vectors transformation and protein expression visualization

The vectors containing the *TaSUMO4-7* genes were introduced into onion epidermal cells with a particle bombardment technique (Biolistic^®^ PDS-1000/He, BioRad, Hercules, CA, USA). The bombarded cells were incubated in the dark for 24 h at room temperature. The expression of the TaSUMO4-7 proteins was then visualized by confocal laser scanning microscopy (FV300-BX61; Olympus, Tokyo, Japan) following protocols explored by Kitajima et al. (2009) and Ikarashi et al. (2012).

## Results

### New *TaSUMO* genes molecular characterization

**Figure 1:**
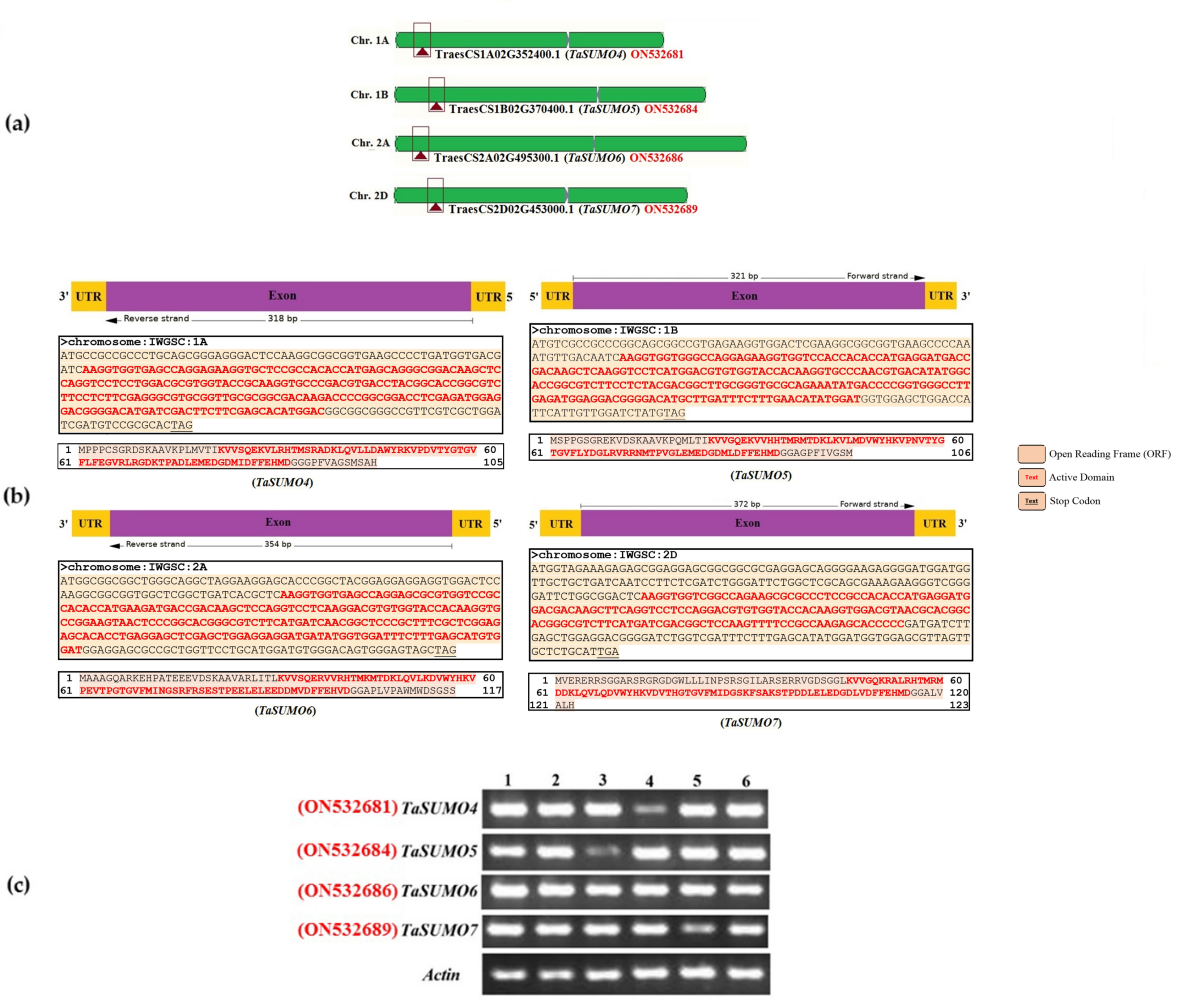
Characterization and expression analysis of *TaSUMO4-7* genes in wheat. (A) Wheat chromosomes and locations of the *TaSUMO4, 5, 6, and 7* genes. (B) *TaSUMO4, 5, 6, and 7* gene structure, coding regions and proteins sequences. (C) Transcripts level of *TaSUMO4, 5, 6, and 7* genes. Each transcript was amplified by RT–PCR. 1: young leaves; 2: roots on young plants; 3: mature leaves; 4: flag leaves; 5: spikelets; 6: seeds. As a control, the transcript of the wheat Actin gene was analysed (bottom panel).

The sequences of the novel *TaSUMO* genes were amplified using PCR with specifically designed primers (presented in Table S1). These primers were created based on the DNA sequences of potential *SUMO* regions identified within the wheat genome database (Fig. 1A). Sequencing results revealed that the 318, 321, 354, and 372 bp fragments of the novel *TaSUMO4-7* genes were located on the long arms of chromosomes 1A, 1B, 2A and 2D, respectively, encoding 105, 106, 117, and 123 amino acid SUMO proteins, respectively (Table 1, Fig. 1B).

**Table 1:** Characterization of the putative proteins of the identified wheat *SUMO* genes.

**TaSUMO**	**GenBank accession**	**No. of AA**	**Gene ID**	**Chr. No.**	**No. Exon**	**CDS length (bp)**	**SUMOplotTM prediction of SUMOylation sites**					
							**Motifs with high probability**			**Motifs with low probability**		
							**Motif**	**Pos.**	**Score**	**Motif**	**Pos.**	**Score**
TaSUMO1	ON532674	101	TraesCS3A02G424000.1	3A	3	743	-	-	-	DKKP	K10	0.39
	ON532675		TraesCS3B02G460000.1	3B		677						
	ON532676		TraesCS3D02G419300.1	3D		851						
TaSUMO2	ON532677	105	TraesCS3B02G459900.1	3B	3	767	-	-	-	DKKP	K10	0.39
	ON532678	106	TraesCS3D02G419200.1	3D		709						
TaSUMO3	ON532679	131	TraesCS3A02G366700.1	3A	1	396	VKPE	K37	0.93	-	-	-
	ON532680	123	TraesCS3D02G359600.1	3D		478						
TaSUMO4	ON532681	105	TraesCS1A02G352400.1	1A	1	318	-	-	-	DKTP RKVP	K72 K50	0.39 0.34
	ON532682	104	TraesCS1B02G369700.1	1B		315				HKVP	K49	0.41
	ON532683	105	TraesCS1D02G357900.1	1D		318				HKVP DKTP	K50 K72	0.41 0.39
TaSUMO5	ON532684	106	TraesCS1B02G370400.1	1B	1	321	-	-	-	EKVD HKVP	K10 K53	0.50 0.41
	ON532685	106	TraesCS1D02G358000.1	1D		321						
TaSUMO6	ON532686	117	TraesCS2A02G495300.1	2A	1	354	-	-	-	HKVP	K59	0.41
	ON532687	113	TraesCS2B02G523500.1	2B		342						
	ON532688	112	TraesCS2D02G495600.1	2D		471						
TaSUMO7	ON532689	123	TraesCS2D02G453000.1	2D	1	372	–	–	–	HKVD	K74	0.52

RT-PCR was employed to assess the expression levels of the *TaSUMO4-7* genes across various wheat tissues, encompassing young leaves, roots from young plants, mature leaves, flag leaves, spikelets, and seeds. The new *TaSUMO*-genes were found to be expressed in all of the wheat tested tissues (Fig. 1C), demonstrating that they are expressed in a constitutive manner.

**Figure 2:**
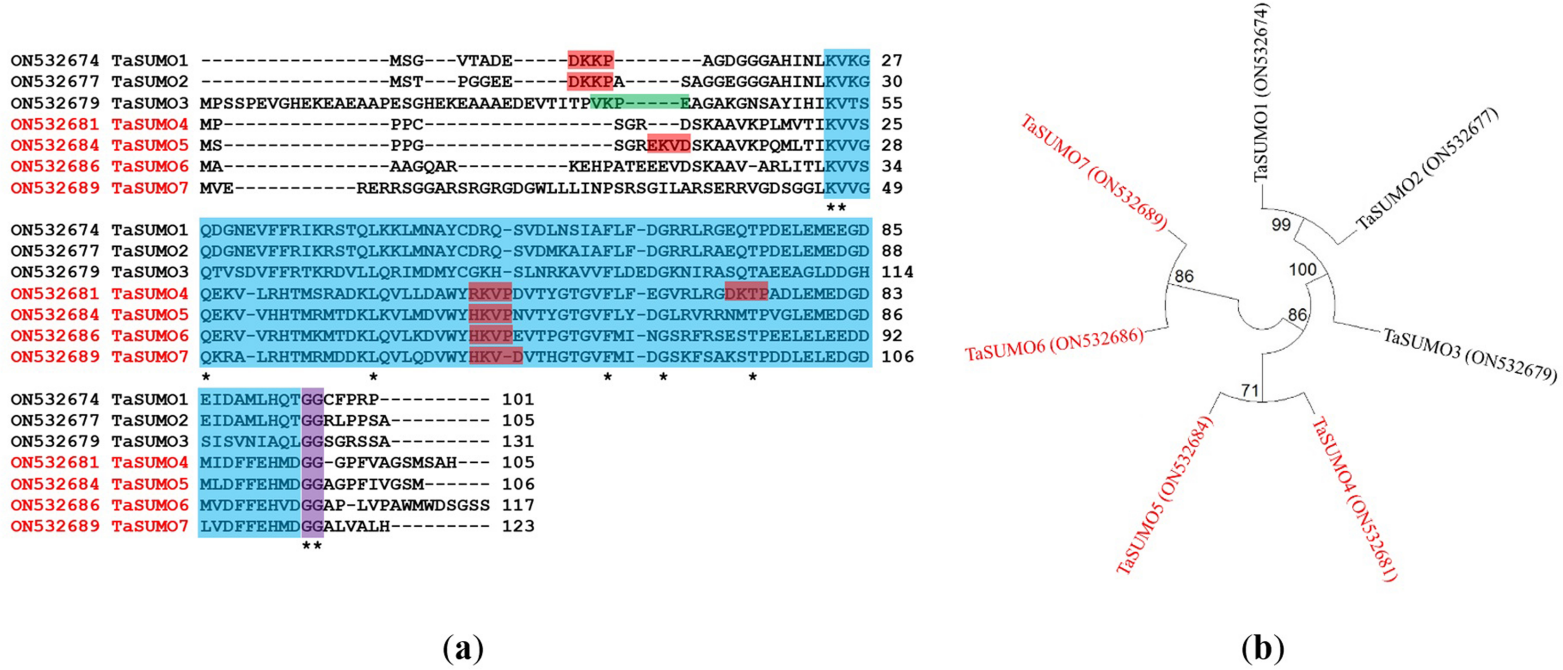
Sequence alignment and phylogenetic analysis of *TaSUMO* genes: identification of conserved Motifs, SUMOylation sites, and evolutionary relationships. (A) *TaSUMO* genes alignment. Di-glycine motifs at the ends of the mature TaSUMO proteins are indicated by the purple box. Potential SUMOylation sites (Ψ KXE/D) are boxed in green for a high probability and with red for a low probability. The cyan box indicates the conserved ubiquitin-like domain. Identical amino acids in TaSUMO proteins are marked with an asterisk. (B) Phylogenetic tree analysis of the TaSUMO family.

Proteins with 105, 106, 117, and 123 amino acid residues were predicted for the unique *TaSUMO4-7* genes according to a BLAST investigation performed built on protein sequences in the wheat genome database. All the identified proteins possessed a C terminal di.glycine (GG) motif and shared consensus motifs (ΨKXE/D), indicative of a high resemblance degree of sequence with other known TaSUMO members (Fig. 2A). These findings revealed that *TaSUMO4-7* contains SUMO protein family features.

**Figure 3:**
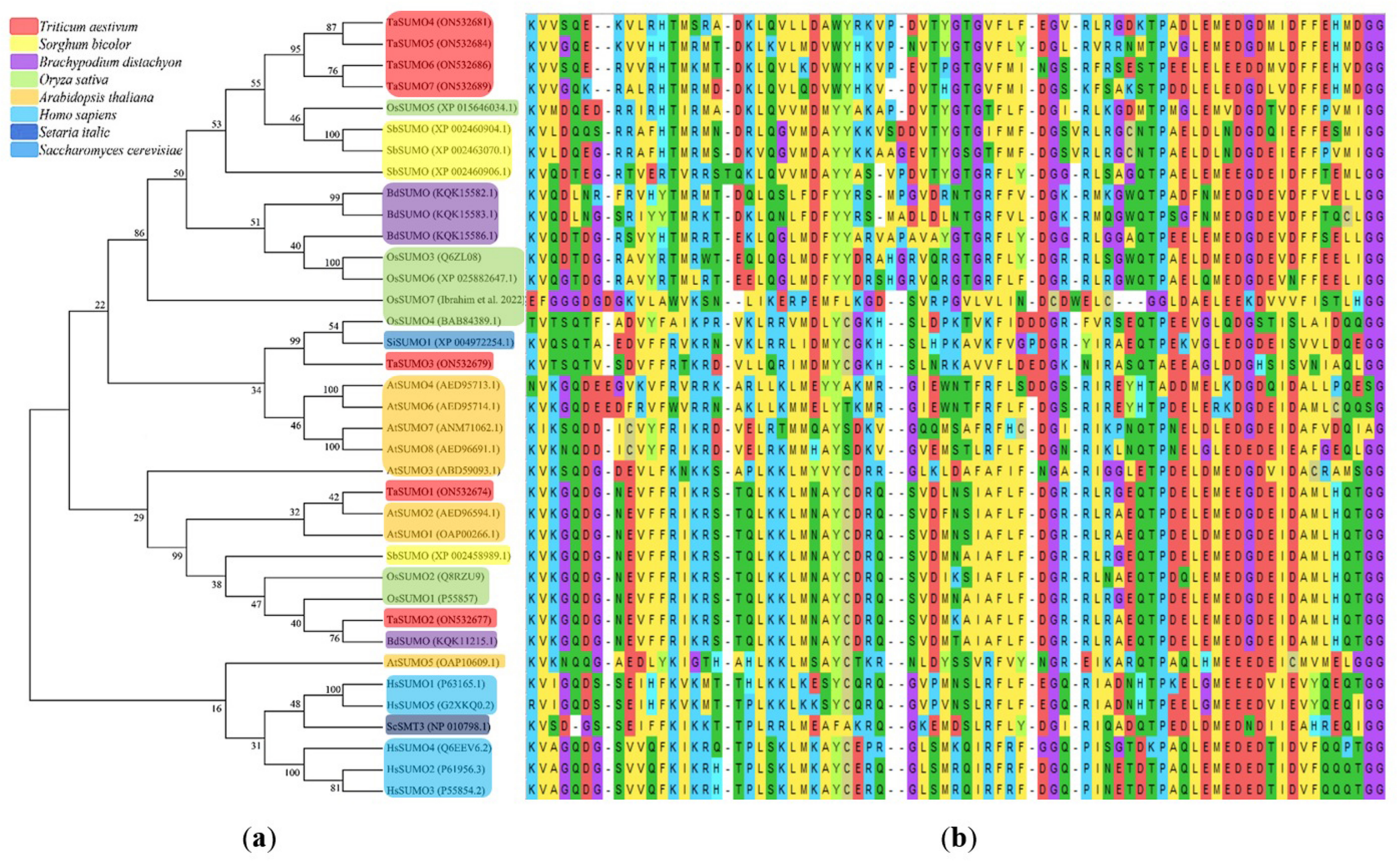
Multiple sequence alignment results of SUMOs from ClustalW. (A) Phylogenetic tree analysis of *SUMO* genes. (B) SUMOs alignment of the conserved ubiquitin-like domain.

A phylogenetic tree was established utilizing SUMO protein sequences from diverse species, encompassing wheat (*Triticum aestivum*, Ta), *Arabidopsis thaliana* (At), rice (*Oryza sativa*, Os), *Brachypodium distachyon* (Bd), human (*Homo sapiens*, Hs), sorghum (*Sorghum bicolor*, Sb), foxtail millet (*Setaria italica*, Si), and baker’s yeast (*Saccharomyces cerevisiae*, Sc). The phylogenetic tree exposed a high similarity degree between the four novel *TaSUMO* genes since they were clustered in the same tree, and the degree of similarity was greater between each of the TaSUMO4 and TaSUMO5 proteins and between the TaSUMO6 and TaSUMO7 proteins because they were found in the same sub-tree (Figs. 2B, 3A).

These findings were supported by the results obtained from Vector NTI^®^ Software (Invitrogen, Waltham, MA, USA) for the percentage of amino acid identity between TaSUMO proteins; however, the percentages were 71% and 72% between TaSUMO4 and TaSUMO5 and between TaSUMO6 and TaSUMO7, respectively (Table 2). Monocotyledons including rice (*Oryza sativa*, Os) and sorghum (*Sorghum bicolor*, Sb) had similarity with TaSUMO4-7 proteins (Fig. 3A).

**Table 2:** Amino acid sequence identities (%) among wheat SUMO proteins were calculated by using the Vector NTI program.

TaSUMO	SUMO1 (Wang et al., 2018)	SUMO2 (Wang et al., 2018)	SUMO3 (Wang et al., 2018)	SUMO4 (Ibrahim, 2022)	SUMO5 (Ibrahim, 2022)	SUMO6 (Ibrahim, 2022)	SUMO7 (Ibrahim, 2022)
SUMO1	100						
SUMO2	84	100					
SUMO3	36	42	100				
SUMO4	38	38	33	100			
SUMO5	36	38	28	71	100		
SUMO6	35	34	24	63	67	100	
SUMO7	31	34	30	64	65	72	100

TaSUMOs and SUMOs from diverse species have similar protein sequences (Fig. 2A) and conserved domains (Fig. 3B), indicating that *TaSUMO4-7* are evolutionarily conserved SUMO family members.

### TaSUMO4-7 *in silico* characterization

TaSUMO4-7 proteins were characterized using bioinformatic analysis and compared with other TaSUMO, AtSUMO and OsSUMO proteins. The *TaSUMO4* and *TaSUMO5* genes were found on the long arm of chromosome one; the *TaSUMO6* and *TaSUMO7* genes on the long arm of chromosome two; and the *TaSUMO1*, *TaSUMO2*, and *TaSUMO3* genes on chromosome three, according to the findings (Table 1). The results showed that the *TaSUMO4-7* genes have one exon, similar to the *TaSUMO3* gene, whereas the *TaSUMO1* and *TaSUMO2* genes have three exons (Table 1). Furthermore, the TaSUMO4 protein has two motifs for SUMOylation sites at the K72 and K50 positions, while TaSUMO5 has two motifs at K10, similar to TaSUMO1, 2 and K53 positions; however, TaSUMO6, 7 have only one motif at the K59 and K74 positions, respectively (Table 1).

### Investigations of biochemical properties

**Figure 4:**
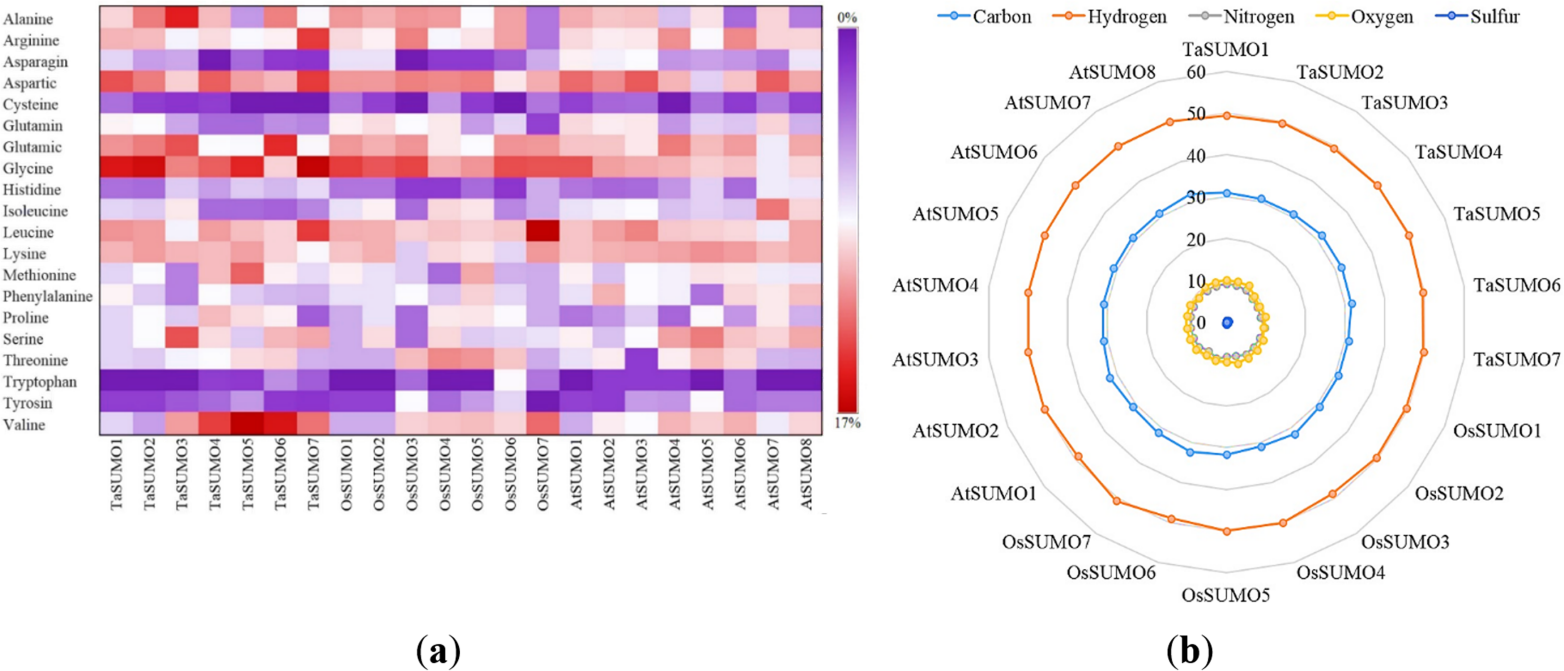
Compositional profiling of TaSUMOs, OsSUMOs, and AtSUMOs: amino acid and atomic distribution patterns. (A) Heatmap demonstrating the amino acid compositions of TaSUMOs, OsSUMOs and AtSUMOs according to percentage. A common amino acid found in all SUMOs is depicted. (B) Radar graph showing the atomic composition by percentage (number of each atom divided by the number of total atoms) of TaSUMOs, OsSUMOs and AtSUMOs.

**Figure 5:**
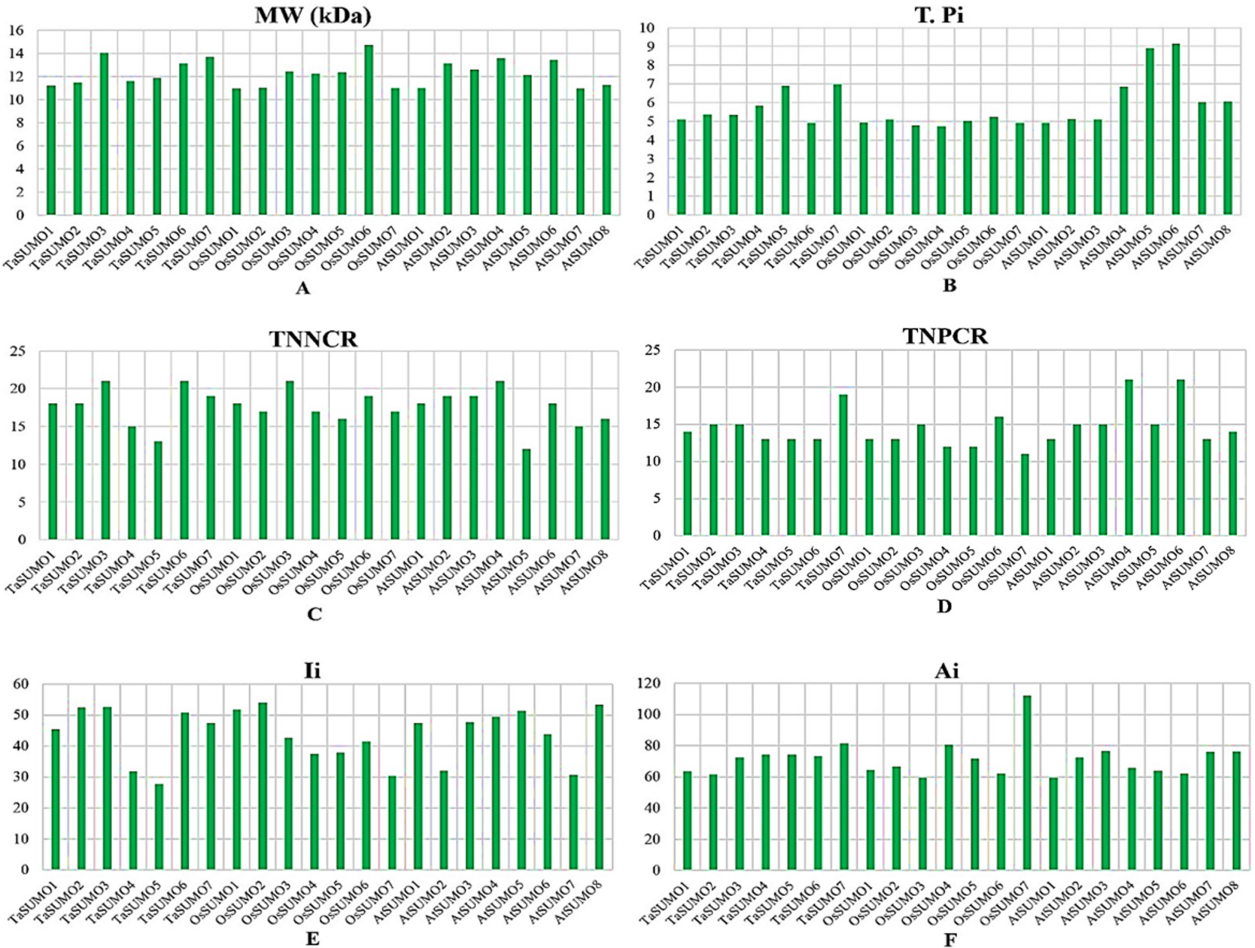
Chart represents the biochemical properties of TaSUMOs, OsSUMOs, and AtSUMOs determined by ProtParam. (A) Molecular weight; (B) Theoretical pI; (C) Total number of negatively charged residues (Asp + Glu); (D) Total number of positively charged residues (Arg + Lys); (E) Ii Instability index; (F) Aliphatic index.

Table 3 and Table S2 and Figs. 4 and 5 show the results of the biochemical features analysis of the SUMO proteins performed by ProtParam. The MWs of the TaSUMO4-7 proteins were determined to be 11.58, 11.84, 13.10, and 13.68 kDa. The MW of the TaSUMO6 protein was the same as the MW of the AtSUMO2 protein. The T-Pi values for the TaSUMO4-7 proteins were 5.85, 6.90, 4.91, and 6.98. The TaSUMO5 protein has a T-Pi value that is similar to that of TaSUMO7 and AtSUMO4.

Furthermore, the TaSUMO4-6 proteins had the same TNPCR (Arg + Lys) values (13) as OsSUMO1, OsSUMO2, AtSUMO1, and AtSUMO1. For TaSUMO5, seven proteins, TNNCR (Asp + Glu) and TNPCR (Arg + Lys), had equal values of 13 and 19, respectively. Moreover, the TaSUMO4 protein, like AtSUMO7, had a TNNCR (Asp + Glu) value (15), and the TaSUMO6 protein had a value (21) similar to that of TaSUMO3, OsSUMO3 and AtSUMO4 for the same parameter. Additionally, the TaSUMO7 protein had a value (19) identical to that of the OsSUMO6, AtSUMO2 and AtSUMO3 proteins (Table 3, Table S2).

The instability index (Ii) values for TaSUMO4 and five proteins were less than 40 (31.91, 27.71), suggesting that the proteins were stable; however, for TaSUMO6, seven proteins were more than 40 (50.80, 47.52), showing that the proteins were unstable, similar to the TaSUMO1-3 proteins. Moreover, the Ai values for the TaSUMO4-6 proteins were fairly comparable (74.19, 74.25, 73.25), whereas the TaSUMO7 protein was slightly different (81.54).

**Table 3:** TaSUMOs biochemical properties determined using ProtParam.

**TaSUMO**	**GenBank accession**	**Formula**	**MW (kDa)**	**T. Pi**	**TNNCR**	**TNPCR**	**Ii**	**Stability**	**Ai**	**GRAVY**
TaSUMO1	ON532674	C_479_H_764_N_142_O_156_S_6_	11.20	5.11	18	14	45.37	Unstable	63.76	−0.75
TaSUMO2	ON532677	C_489_H_788_N_146_O_160_S_6_	11.46	5.36	18	15	52.46	Unstable	61.43	−0.77
TaSUMO3	ON532679	C_599_H_964_N_176_O_203_S_4_	14.01	5.33	21	15	52.61	Unstable	72.29	−0.53
TaSUMO4	ON532681	C_512_H_809_N_139_O_151_S_8_	11.58	5.85	15	13	31.91	Stable	74.19	−0.21
TaSUMO5	ON532684	C_524_H_832_N_142_O_150_S_10_	11.84	6.90	13	13	27.71	Stable	74.25	−0.21
TaSUMO6	ON532686	C_576_H_903_N_157_O_180_S_6_	13.10	4.91	21	13	50.80	Unstable	73.25	−0.40
TaSUMO7	ON532689	C_591_H_952_N_184_O_180_S_5_	13.68	6.98	19	19	47.52	Unstable	81.54	−0.49

**Notes.**

MW Molecular weight T. Pi Theoretical pI TNNCR Total number of negatively charged residues (Asp + Glu) TNPCR Total number of positively charged residues (Arg + Lys) Ii Instability index Ai Aliphatic index GRAVY Grand average of hydropathicity

In terms of GRAVY, TaSUMO4 and TaSUMO5 proteins had equal values (−0.21), while TaSUMO6 and TaSUMO7 proteins had extremely close values (−0.40 and −0.49, respectively), as shown in Table 3 and Table S2. The amino acid composition analysis revealed that the TaSUMO5-7 proteins sequences had all 20 amino acids except cysteine (C), whereas the TaSUMO4 protein sequences contained all 20 amino acids except asparagin (N) (Fig. 4A, Table S3). Among the analysed SUMO proteins, TaSUMO4 had the highest percentage of the amino acid Pro (P, 6.7%), while the TaSUMO5 protein had the highest percentage of the amino acids Met (M, 9.4%) and Val (V, 13.2%). Additionally, TaSUMO6 had the largest amount of the amino acid Glu (E, 11.1%) and the lowest percentage of the amino acid Ile (I, 1.7%).

Additionally, the TaSUMO7 protein possessed high proportions of Arg (R, 10.6%) and Gly (G, 13%) amino acids and the lowest ratio of Pro (P, 1.6%) amino acid. Among the basic 20 amino acids, valine was the component with the largest percentage (10.2%, 13.2%, 12%) of the TaSUMO4-6 proteins, while it was a glycine (13%) in the TaSUMO7 protein (Fig. 4A, Table S4).

The amino acid’s atomic composition (AC) includes (carbon, C), (hydrogen, H), (nitrogen, N), (oxygen, O), and (sulfur, S). OsSUMO6 had the highest TNA value (2,034) among the analysed SUMO proteins, while TaSUMO3 had the highest TNA value (1,946) among the TaSUMO proteins, and TaSUMO7 had the highest TNA value among the four novel wheat SUMO proteins (Table S3). Analysis of the atomic composition using a radar graph (Fig. 4B) revealed a highly conserved elemental profile across all tested SUMO proteins from wheat, rice, and Arabidopsis, with only minor visual variations.

### Secondary and tertiary structure prediction of TaSUMO4-7 proteins

**Figure 6:**
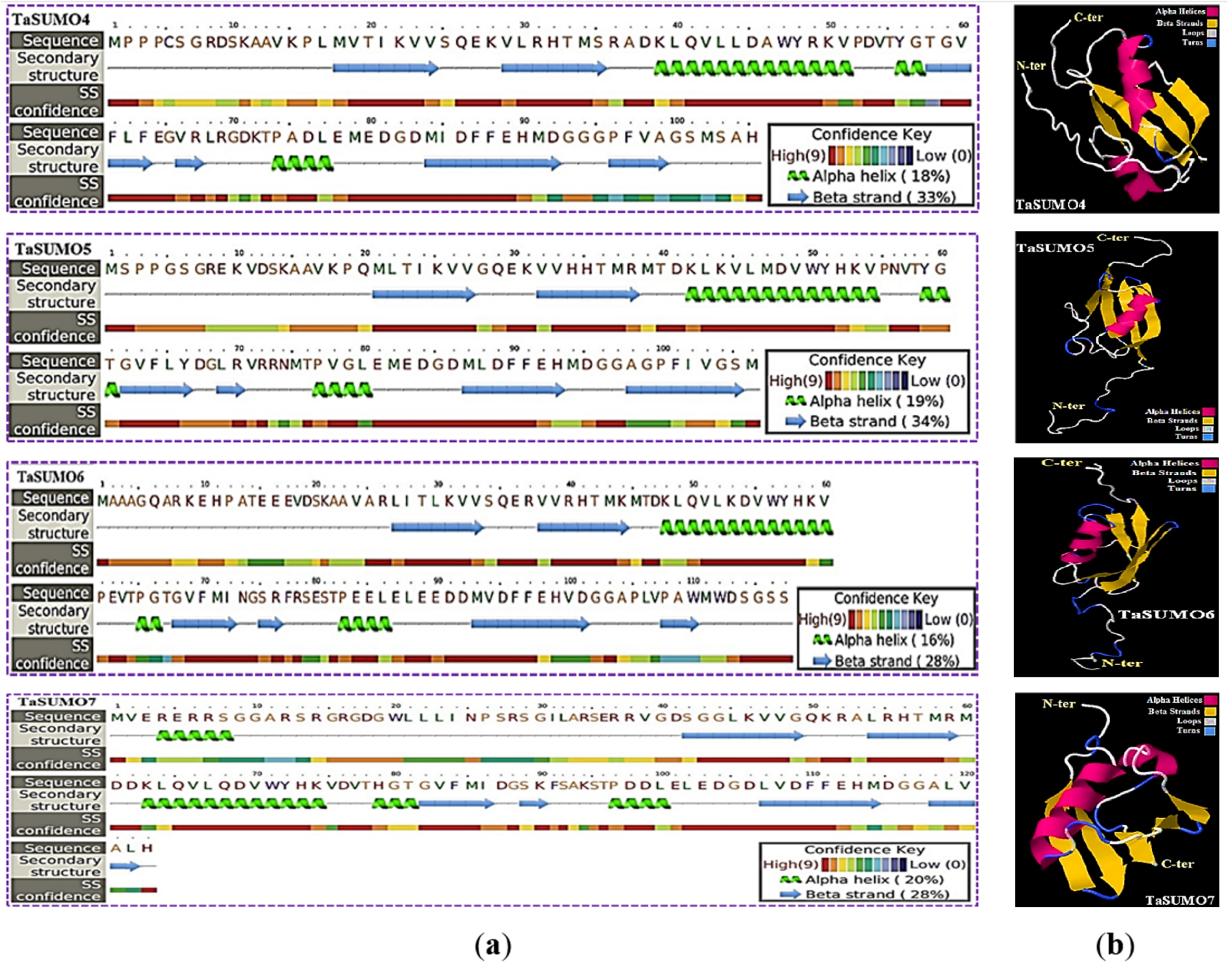
Structural prediction and 3D modeling of TaSUMO4, TaSUMO5, TaSUMO6, and TaSUMO7 proteins using Phyre2. (A) Prediction of the secondary structure of the putative TaSUMO4, 5, 6, and 7 proteins by the Phyre2 server. (B) The predicted 3D model of the TaSUMO4, 5, 6, and 7 proteins, designed by the Phyre2 server.

The secondary structure was obtained using the PSIPRED method (Jones, 1999), which is based on sequence-based prediction. The predicted secondary structure of the protein contained three main states: α-helices, β-strands, and coils. These are visually represented by green helices, blue arrows, and faint lines, respectively. The confidence level in the prediction is also displayed, with red representing high confidence and blue demonstrating low confidence. The predicted secondary structure of all the novel TaSUMO proteins included six β-strands intervened by nine coils and three helixes, with the exception of TaSUMO7, which has four helixes (Fig. 6A). Using the Phyre2 engine, the three-dimensional model of TaSUMO5,6 were obtained based on the crystal structure of the human (*H. sapiens*) SUMO-1 domain (PDB code 1A5R) with a confidence of 99.9%, whereas the 3D structure of the TaSUMO4 protein was determined based on the crystal structure of the *Trypanosoma brucei* SUMO chain (PDB code 2K8H) with a confidence of 99.8%. However, for the TaSUOM7 protein, the 3D structural model was established by relying on the crystal structure of the *Mus musculus* SUMO chain (PDB code 3A4R) with a confidence of 99.7%. JSmol (JavaScript-Based Molecular Viewer From Jmol) (Hanson et al., 2013) was used to view 3D structural models of proteins (Fig. 6B).

### TaSUMO4-7 proteins cellular localization and expression

**Figure 7:**
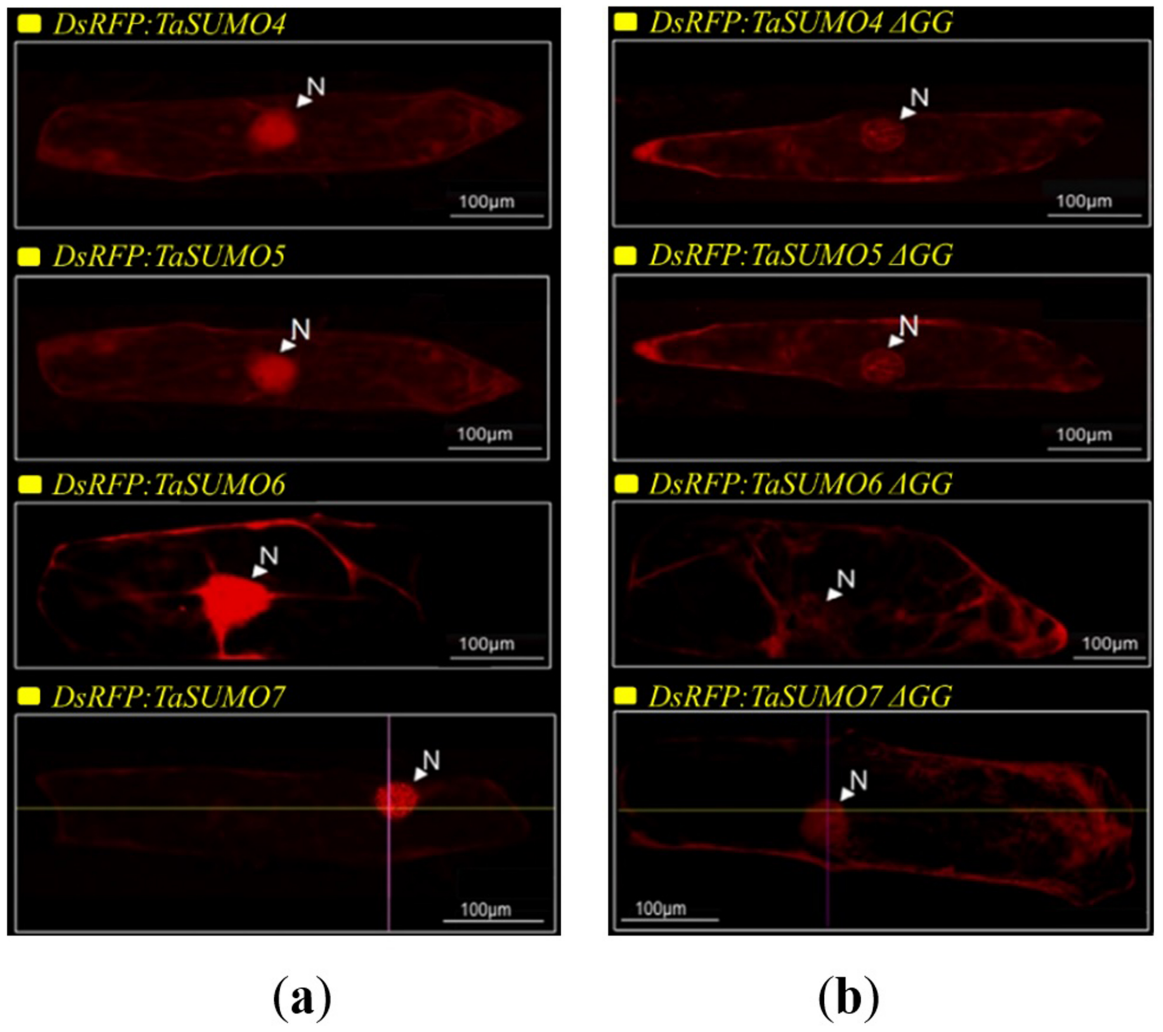
Cellular localization of TaSUMO4-7 proteins in onion cells: role of the GG motif in subcellular targeting. (A) Localization of *DsRFP:TaSUMO4,5,6,7* in onion cells. (B) Localization of *DsRFP:TaSUMO4,5,6,7* with a GG deletion in onion cells.

**Figure 8:**
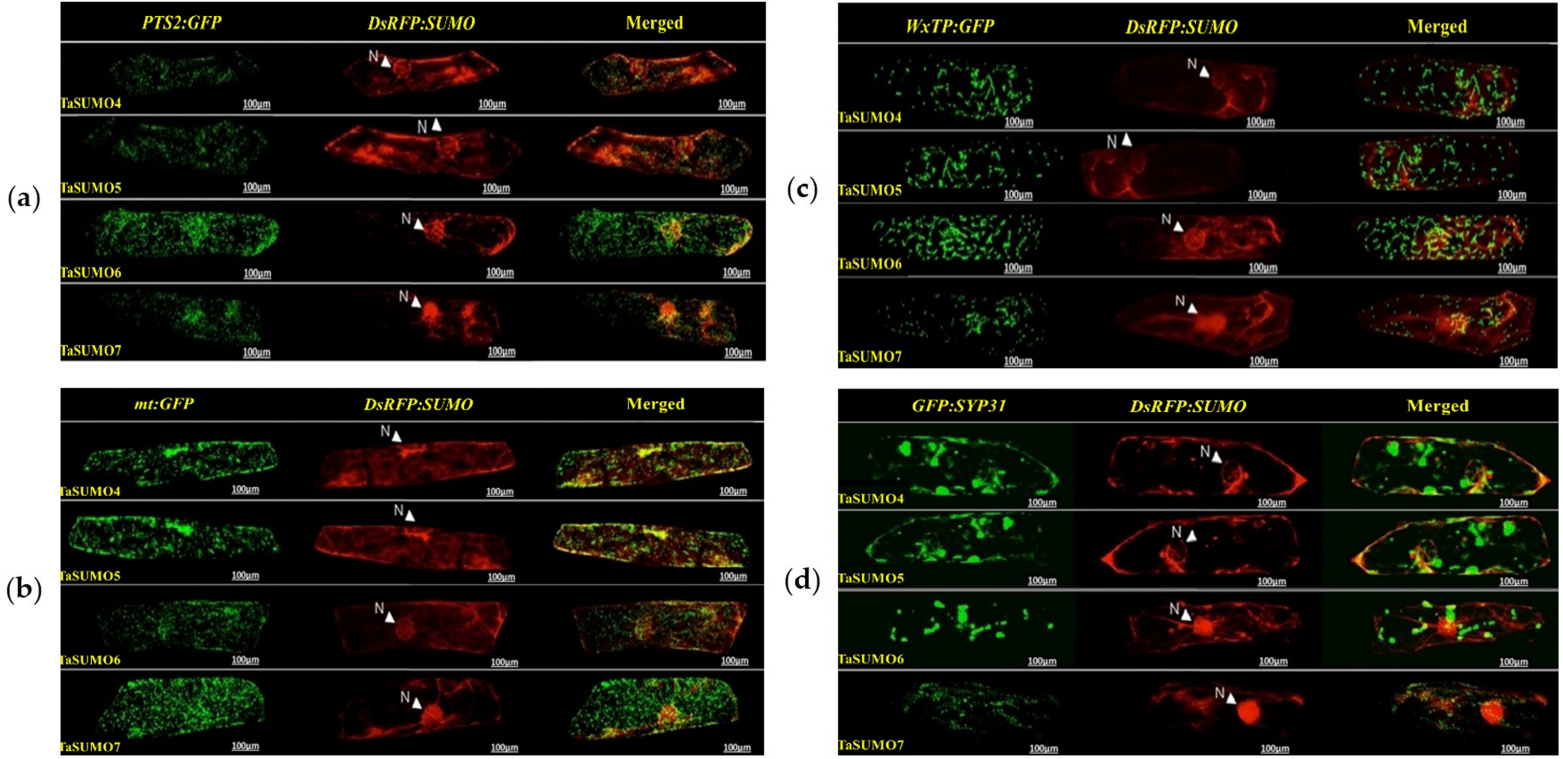
Organelle-specific localization of TaSUMO4-7 proteins in onion cells: insights into peroxisomal, mitochondrial, plastidial, and Golgi targeting. (A) Coexpression of *DsRFP:TaSUMO4,5,6,7* and *PTS2:GFP* in onion cells. PTS2 peroxisomal marker. (B) Coexpression of *DsRFP:TaSUMO4,5,6,7* and *mt:GFP* in onion cells. mt; mitochondrial marker. (C) Coexpression of *DsRFP:TaSUMO4,5,6,7* and *WxTP:GFP* in onion cells. WxTP, plastidial marker. (D) Coexpression of *DsRFP:TaSUMO4,5,6,7* and *SYP31:GFP* in onion cells. SYP31, cis-Golgi marker.

*DsRFP:TaSUMO4*, *DsRFP:TaSUMO5* and *DsRFP:TaSUMO6* are found mainly in the cytoplasm and nucleus of onion cells, while *DsRFP:TaSUMO7* is mostly localized in the nucleus (Fig. 7A). Additionally, the fusion proteins *DsRFP:TaSUMO4*Δ*GG, DsRFP:TaSUMO5*Δ*GG, DsRFP:TaSUMO6*Δ*GG,* and *DsRFP:TaSUMO7*Δ*GG* were observed in both the nucleus and cytoplasm, with significantly weaker signals in the nucleus (Fig. 7B). To further investigate the localization of these proteins, we used vectors expressing fluorescent markers (*pPTS2:GFP, pmt:GFP, pWxTP:GFP, pGFP:SYP31*) for different organelles (peroxisomes, mitochondria, plastids, and Golgi apparatuses). This allowed us to determine whether the SUMO proteins localize to any other cellular compartments. In onion epidermal cells, *pDsRFP:TaSUMO4, pDsRFP:TaSUMO5, pDsRFP:TaSUMO6* and *pDsRFPd:TaSUMO7* were transfected into cells. According to our findings, TaSUMO4-7 proteins were not found among any of the previously mentioned organelles under investigation (Fig. 8).

### *GFP:SCE1a* and TaSUMO4-7 co localization and co expression

**Figure 9:**
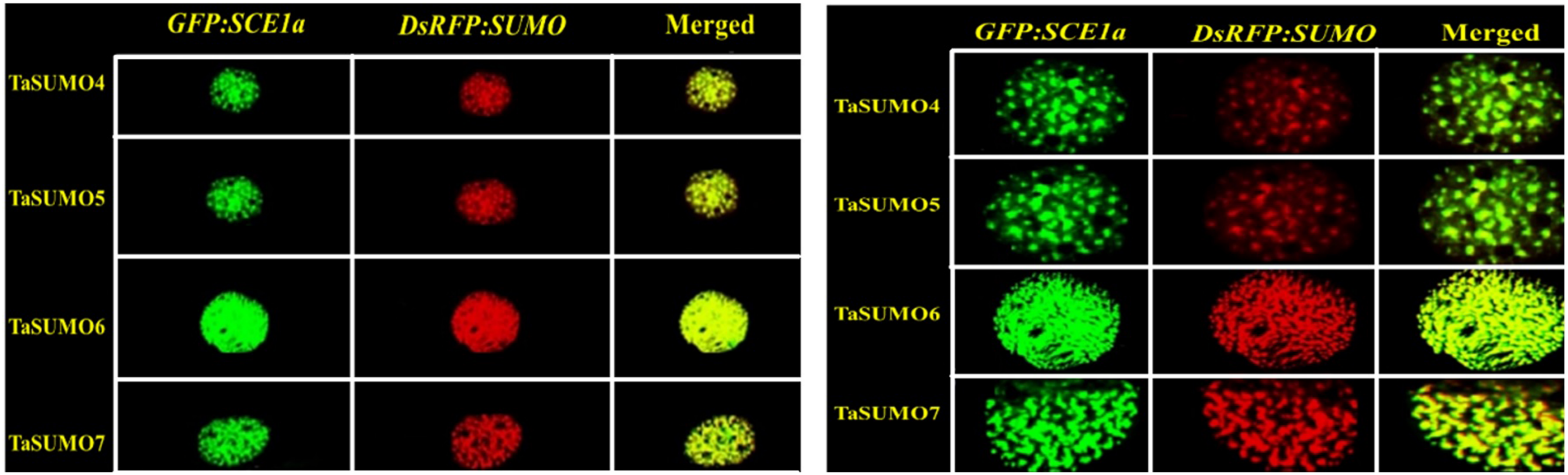
Nuclear co-localization of DsRFP-Tagged TaSUMO4, TaSUMO5, TaSUMO6, and TaSUMO7 with GFP:SCE1a in onion epidermal cells. (A) Co-expression and co-localization of *DsRFP:TaSUMO4,5,6,7* with *GFP:SCE1a* in the nucleus of onion cells. (B) Co-expression and co-localization of *DsRFP:TaSUMO4,5,6,7* with *GFP:SCE1a* in the nucleus of onion cells at high magnification.

The *GFP:SCE1a* (SUMO-conjugation-enzyme) and *DsRFP:TaSUMO4-7* fusion proteins colocalized and coexpressed in the nucleus, where they formed nuclear sub-domains. Unlike signals observed when expressed alone, signals were collected as dot-like structures inside the nucleus (Fig. 9A). In addition, high-magnification observation was performed to analyse the localization of the proteins in detail. High-magnification imaging (Fig. 9B) revealed colocalization of *DsRFP*-tagged TaSUMO4-7 proteins with the SUMO-conjugating enzyme *GFP:SCE1a* within the nucleus, suggesting that conjugation of SUMO proteins to their substrates likely occurs at these sites. However, it is important to note that the processing of the C-terminal di-glycine motif, essential for conjugation, may have already taken place in other cellular compartments prior to nuclear localization.

## Discussion

This study identified and characterized four novel *SUMO* genes within the wheat genome, named *TaSUMO4*, *TaSUMO5*, *TaSUMO6*, and *TaSUMO7*, representing the first discovery of these genes in wheat. The sequencing analysis of the putative proteins of these genes demonstrated to have the SUMOylation features, including a C terminal diglycine (GG) motif and consensus motifs (ΨKXE/D), indicating a high degree of homology to other *TaSUMO* members (Fig. 2A). Many *SUMO*-genes have been described in several plant species, including rice (Ikarashi et al., 2012; Rosa et al., 2018; Ibrahim et al., 2021; Teramura et al., 2021; Ibrahim, 2022) and Arabidopsis (Kurepa et al., 2003). However, only one study of *SUMO*-genes in wheat, in which three genes were identified and characterized *in-silico*, has been found in the database (Mayer et al., 2014; Wang et al., 2018). Our findings defined four genes and were built on a previous report in the nomenclature and comparison.

The phylogenetic tree showed a high homology degree among the four novel *TaSUMO* genes, and there was a close similarity between *TaSUMO4* and *TaSUMO5*, and between *TaSUMO6* and *TaSUMO7* (Figs. 2B, 3A). Coincidentally, monocotyledons including (*Oryza sativa*, Os) and (*Sorghum bicolor*, Sc) showed high resemblance with TaSUMO4-7 proteins (Fig. 3A), which might share several important functions. The study revealed that the newly discovered TaSUMO4-7 proteins are evolutionarily well-preserved members of the SUMO family and exhibit close similarity to their counterparts in other species. Evolutionary tree analysis shown that the homologous genes in wheat, Arabidopsis, and rice are more closely related at the proximal level (Wang et al., 2018; Ibrahim et al., 2021). The current study revealed that the *TaSUMO4* and *TaSUMO5* genes were found on the long arm of chromosome one, while the *TaSUMO6* and *TaSUMO7* genes were mapped on the long arm of chromosome two. However, the *TaSUMO1*, *TaSUMO2*, and *TaSUMO3* genes are located on chromosome three (Table 1) (Wang et al., 2018). These results suggested that the identified *TaSUMO*-genes might be distributed and clustered as tandem sets in the wheat genome.

Like findings in Arabidopsis (Hammoudi et al., 2016; Joo et al., 2020) and rice (Wang et al., 2018), this study identified tandem gene pairs among the newly discovered wheat SUMO genes (*TaSUMO4-7*). This finding suggested that tandem duplication, a well-established mechanism for generating genetic diversity and novel gene functions (Lallemand et al., 2020; Ohno, 2013), plays a similar role in the diversification of *SUMO* genes across diverse plant species. The study revealed that the new *TaSUMO4-7* genes have one exon, similar to the *TaSUMO3* gene, whereas the *TaSUMO1* and *TaSUMO2* genes have three exons (Table 1). Furthermore, the *TaSUMO4-7* proteins have different SUMOylation site motifs at different positions, which indicates that these genes are similar and have several functions. These results are consistent with the observations documented in previous studies by Wang et al. (2018), Miura & Hasegawa (2010), and Ibrahim et al. (2021). The transcriptional levels of *TaSUMO4-7* were detected in diverse wheat tissues, including roots of young plants, young leaves, mature leaves, seeds, spikelets, and flag leaves (Fig. 1C), indicating that these genes are constitutively expressed. While our study suggested the ubiquitous expression of *TaSUMO4-7*, previous research by Choulet et al. (2014) and Wang et al. (2018) reported tissue- and time-specific expression patterns for *TaSUMO1-3* genes, highlighting potential functional diversity within the wheat SUMO family. In contrast to our findings, Teramura et al. (2021) reported tissue-specific expression of *OsSUMO4/6* genes in rice, suggesting functional diversification among individual SUMO family members.

This finding aligns with the essential roles of SUMOylation in various cellular processes like hormone signalling, stress response, and development (Rodriguez, Dargemont & Hay, 2001; Srivastava, Zhang & Sadanandom, 2016; Augustine & Vierstra, 2018). Therefore, understanding the expression patterns and cellular localization of *SUMO* transcripts is crucial for deciphering the functional mechanisms of plant SUMOylation. This study explored the subcellular localization of newly discovered TaSUMO proteins in onion cells. *DsRFP-tagged TaSUMO4, 5,* and *6* proteins were found in both the nucleus and cytoplasm, while *TaSUMO7* localized primarily to the nucleus in subdomain structures (Fig. 7A), consistent with previous reports (Lois, Lima & Chua, 2003). These outcomes suggest that the SUMO-activating system processes, activates, and conjugates these proteins within the cell. Removal of the GG motif reduced the nuclear and cytoplasmic accretion of *DsRFP-tagged TaSUMO4-7*Δ*GG* proteins and resulted in weakened nuclear signals (Fig. 7B), highlighting the crucial role of the GG motif in SUMO protein processing, activation, and accumulation. Similar findings have been stated elsewhere (Murtas et al., 2003; Ikarashi et al., 2012; Ibrahim et al., 2021). Additionally, our study revealed no localization of the TaSUMO4-7 proteins to other cell organelles (Fig. 8). Previous research has shown that rice OsSUMO1/2/3 proteins are solely nuclear in rice root cells, while OsSUMO4 is localized to specific nuclear compartments compared to GFP alone (Joo et al., 2020). These results underscore the significance of SUMO proteins in mediating protein interactions, localization, and function through the SUMOylation process.

The SUMO modification system includes SUMO modified substrates, activation enzymes (E1), binding enzymes (E2), ligases (E3), and proteases of SUMO modifiers (Miura et al., 2007b; Park et al., 2011). The SUMO-conjugation enzyme (*GFP:SCE1a*) and *DsRFP:TaSUMO4-7* fusion proteins were colocalized and coexpressed in the nucleus, where they formed nuclear sub-domains (Fig. 9A). These results suggest that *DsRFP:TaSUMO4-7* are processed, activated, conjugated with *GFP:SCE1a*, and transported to localize in nuclear foci *via* the SUMOylation process in onion cells, where it may bind to protein substrates. The wheat genome, like the rice and Arabidopsis genomes, encodes the activation enzymes SAE1a, SAE2, and SAE1B (Wang et al., 2018). Wheat and rice harbor three gene conjugation enzymes, namely, SCE1, SCE2 and SCE3, in addition to the four gene ligation enzymes SIZ1, SIZ2, SIZ3, and SIZ4 (Kurepa et al., 2003; Jin et al., 2008; Castaño-Miquel, Seguí & Lois, 2011; Cai et al., 2017; Wang et al., 2018). Although the conjugation enzyme-encoding genes SCE1, SCE2, and SCE3 have been extensively studied (Chaikam & Karlson, 2010; Joo et al., 2019), they have not yet been well characterized in wheat.

This study pioneered the investigation of the functional localization of TaSUMO4-7 using the *SCE1:GFP* fusion proteins, revealing its presence in distinct nuclear compartments. The SCE1 gene has been stated to show a powerful role in promoting SUMO conjugation to target proteins through the SUMOylation system (Nurdiani, Widyajayantie & Nugroho, 2018; Ibrahim et al., 2021). Increased levels of SUMO-conjugated-proteins (SUMO-conjugates) are hypothesized to play a role in how plants respond to challenges from their environment (abiotic stress) and other organisms (biotic stress), as evidenced by previous research (Castro et al., 2016; Joo et al., 2019). Maturation, activation, binding, connection, and function are all part of the multi-gene biological process produced by SUMO-isoform modification (Park et al., 2011; Wang et al., 2018). Plants and humans contain many SUMO isoforms, unlike yeast (Gareau & Lima, 2010; Novatchkova et al., 2012). In humans, variations in SUMO-isoform expression levels are thought to contribute to their functional diversity (Wang, Peterson & Loring, 2014). While many plant genomes, including wheat, exhibit SUMO gene diversification, the specific functions of these genes in wheat have largely not been explored.

While this study successfully identified four novel *TaSUMO* genes, some limitations should be noted. First, the functional characterization was limited to *in vitro* localization studies, and future work should include in planta validation through transgenic approaches. Second, the expression profiles were examined under normal growth conditions; abiotic/biotic stress treatments could reveal additional regulatory roles. Finally, the interaction partners of these novel TaSUMOs remain to be identified through proteomic approaches. Future studies should focus on (1) generating knockout mutants to determine phenotypic consequences, (2) examining SUMOylation dynamics under stress conditions, and (3) identifying substrate proteins through mass spectrometry-based approaches.

## Conclusions

This study identified and characterized four novel *SUMO* genes (*TaSUMO4-7*) in the wheat genome, confirming their SUMOylation features. All four genes exhibited constitutive expression across wheat tissues. DsRFP-tagged TaSUMO4, 5, and 6 proteins were detected in both the nucleus and cytoplasm, while TaSUMO7 was exclusively expressed in the nucleus. The presence of essential GG motifs in these genes suggested their involvement in SUMO protein processing, activation, and accumulation. Furthermore, the study demonstrated that TaSUMO4-7 proteins undergo processing, activation, conjugation with the *SCE1a* gene, and localization in nuclear foci through the plant cell’s SUMOylation system, indicating their successful processing, activation, and nuclear transport within the plant cell. Although limited to *in vitro* characterization, this study provides the foundation for future investigations into the stress-responsive roles and molecular mechanisms of these newly identified *TaSUMO* genes in wheat.

##  Supplemental Information

10.7717/peerj.20432/supp-1 Supplemental Information 1 Sequences of the primers which have been used in the study

10.7717/peerj.20432/supp-2 Supplemental Information 2 Biochemical Properties of OsSUMOs and AtSUMOs by ProtParam MW, molecular weight; T. Pi, theoretical pI; TNNCR, total number of negatively charged residues (Asp + Glu); TNPCR, total number of positively charged residues (Arg + Lys) ; Ii, instability index; Ai, aliphatic index; and GRAVY, grand average of hydropathicity

10.7717/peerj.20432/supp-3 Supplemental Information 3 Amino Acid and Atomic composition of TaSUMOs, OsSUMOs and AtSUMOs AC Atomic composition; C* Carbon; H* Hydrogen; N* Nitrogen; O* Oxygen; S* Sulfur; TNA Total number of atoms; AAC Amino acid composition; A Alanine; R Arginine; N Asparagin; D Aspartic; C Cysteine ; Q Glutamin; E Glutamic; G Glycine; H Histidine; I Isoleucine; L Leucine; K Lysine; M Methionine; F Phenylalanine; P Proline; S Serine; T Threonine; W Tryptophan; Y Tyrosin; V Valine; NAA Number of amino acids

10.7717/peerj.20432/supp-4 Supplemental Information 4 Amino acid composition of TaSUMOs, OsSUMOs and AtSUMOs by percentage A Alanine; R Arginine; N Asparagin; D Aspartic; C Cysteine; Q Glutamin; E Glutamic; G Glycine; H Histidine; I Isoleucine; L Leucine; K Lysine; M Methionine; F Phenylalanine; P Proline; S Serine; T Threonine; W Tryptophan; Y Tyrosin; V Valin

10.7717/peerj.20432/supp-5 Supplemental Information 5 Plasmid Construction for TaSUMO4-7, GG Deletion Mutants, Organelle Markers, and *SCE1a* Expression in Functional Analyses (a) Construction of expression plasmid harboring *TaSUMO4, 5, 6, 7* gene s. (b) Construction of expression plasmid harboring *TaSUMO4, 5, 6, 7* gene with GG Deletion. (c) Construction of expression plasmid *PTS2:GFP, mt:GFP, WxTP:GFP, SYP31:GFP*. (d) Construction of expression plasmid harboring* SCE1a* gene

10.7717/peerj.20432/supp-6 Supplemental Information 6 Sequences

## Additional Information and Declarations

### Data Availability

The following information was supplied regarding data availability: The SUMO genomic/cDNA/protein sequences are available at NCBI:
ON640912, ON640913, ON640914, ON640915, ON640916, ON640917, ON640918, ON640919, ON640920, ON640921, ON640922, ON640923, ON640924, ON640925, ON640926, ON640927, ON532674, ON532675, ON532676, ON532677, ON532678, ON532679, ON532680, ON532681, ON532682, ON532683, ON532684, ON532685, ON532686, ON532687, ON532688, ON532689, WAK97207, WAK97208, WAK97209, WAK97210, WAK97211, WAK97212, WAK97213, WAK97214, WAK97215, WAK97216, WAK97217, WAK97218, WAK97219, WAK97220, WAK97221, WAK97222.
