# Supplementary material for: Identification and characterization of novel SUMO genes in bread wheat
Source: PeerJ. 2025 Nov 28;13:e20432. doi: 10.7717/peerj.20432 (PMC12667693; doi:10.7717/peerj.20432)
Supplement: Supplemental Information 1 [file peerj-13-20432-s001.docx]

| **Primer name** | **Primer sequence** |
| --- | --- |
| DsRed T-de F ^(Ikarashi et al. 2012)^ | 5' GTTCCTGAGCGGCCGCATGT 3' |
| DsRed T-de R ^(Ikarashi et al. 2012)^ | 5' GGCCGCTCAGGAACAGGTGG 3' |
| cDNA DsRed T-de F ^(Ikarashi et al. 2012)^ | 5' GTTCCTGTAGCGGCCGCATGT 3' |
| cDNA DsRed T-de R ^(Ikarashi et al. 2012)^ | 5' GGCCGCTACAGGAACAGGTGG 3' |
| TaSUMO4 F ^(Ibrahim 2022)^ | 5' CGAACTCCACGGATCTCAC '3 |
| TaSUMO4 R ^(Ibrahim 2022)^ | 5' TCCCTCTCATCAATCCTGCC '3 |
| RT- TaSUMO4 F ^(Ibrahim 2022)^ | 5′ TGAAGCCCCTGATGGTGAC ′3 |
| RT- TaSUMO4 R ^(Ibrahim 2022)^ | 5′ AGAAGTCGATCATGTCCCCG ′3 |
| TaSUMO5 F ^(Ibrahim 2022)^ | 5' AGGCAGGTACACAACGGAAG 3' |
| TaSUMO5 R ^(Ibrahim 2022)^ | 5' GCTTCAAGTTACAACAACTCCG 3' |
| RT- TaSUMO5 F ^(Ibrahim 2022)^ | 5' AGAAGGTGGACTCGAAGGC 3' |
| RT- TaSUMO5 R ^(Ibrahim 2022)^ | 5' GTCCAGCTCCACCATCCATA 3' |
| TaSUMO6 F ^(Ibrahim 2022)^ | 5' TCCAGATTCCAGAACAACAACAC 3' |
| TaSUMO6 R ^(Ibrahim 2022)^ | 5' CATAGCGGCGAAGAGGTTC 3' |
| RT- TaSUMO6 F ^(Ibrahim 2022)^ | 5' CTGGGCAGGCTAGGAAGG 3' |
| RT- TaSUMO6 R ^(Ibrahim 2022)^ | 5' TAGCTACTCCCACTGTCCCA 3' |
| TaSUMO7 F ^(Ibrahim 2022)^ | 5' CTACGGAGGGCGTCGATG 3' |
| TaSUMO7 R ^(Ibrahim 2022)^ | 5' CAATATCCGGGCGAAGAGGT 3' |
| RT- TaSUMO7 F ^(Ibrahim 2022)^ | 5' TGGTAGAAAGAGAGCGGAGG 3' |
| RT- TaSUMO7 R ^(Ibrahim 2022)^ | 5' AACTARACGCTCCACCATCCA 3' |
