# Supplementary material for: Identification and characterization of novel SUMO genes in bread wheat
Source: PeerJ. 2025 Nov 28;13:e20432. doi: 10.7717/peerj.20432 (PMC12667693; doi:10.7717/peerj.20432)
Supplement: Supplemental Information 2 — MW, molecular weight; T. Pi, theoretical pI; TNNCR, total number of negatively charged residues (Asp + Glu); TNPCR, total number of positively charged residues (Arg + Lys) ; Ii, instability index; Ai, aliphatic index; and GRAVY, grand average of hydropathicity [file peerj-13-20432-s002.docx]

| **SUMOs** | **Formula** | **MW (kDa)** | **T. Pi** | **TNNCR** | **TNPCR** | **Ii** | **Stability** | **Ai** | **GRAVY** |
| --- | --- | --- | --- | --- | --- | --- | --- | --- | --- |
| OsSUMO1 | C_464_H_744_N_138_O_153_S_7_ | 10.93 | 4.95 | 18 | 13 | 51.75 | Unstable | 64.5 | −0.681 |
| OsSUMO2 | C_471_H_756_N_138_O_156_S_5_ | 11.01 | 5.1 | 17 | 13 | 54.05 | Unstable | 66.73 | −0.721 |
| OsSUMO3 | C_542_H_834_N_156_O_173_S_4_ | 12.43 | 4.77 | 21 | 15 | 42.58 | Unstable | 59.45 | −0.718 |
| OsSUMO4 | C_532_H_859_N_145_O_176_S_5_ | 12.26 | 4.73 | 17 | 12 | 37.37 | Stable | 80.44 | −0.264 |
| OsSUMO5 | C_545_H_858_N_142_O_164_S_10_ | 12.34 | 5.01 | 16 | 12 | 37.87 | Stable | 71.73 | −0.174 |
| OsSUMO6 | C_657_H_995_N_183_O_195_S_4_ | 14.71 | 5.23 | 19 | 16 | 41.52 | Unstable | 62.31 | −0.605 |
| OsSUMO7 | C_492_H_795_N_127_O_146_S_5_ | 10.99 | 4.92 | 17 | 11 | 30.35 | Stable | 111.9 | 0.031 |
| AtSUMO1 | C_462_H_740_N_140_O_158_S_6_ | 10.98 | 4.91 | 18 | 13 | 47.48 | Unstable | 59.60 | −0.84 |
| AtSUMO2 | C_578_H_903_N_159_O_177_S_6_ | 13.10 | 5.13 | 19 | 15 | 31.96 | Stable | 72.33 | −0.47 |
| AtSUMO3 | C_550_H_866_N_154_O_170_S_7_ | 12.58 | 5.09 | 19 | 15 | 47.57 | Unstable | 76.49 | −0.58 |
| AtSUMO4 | C_586_H_939_N_173_O_185_S_5_ | 13.53 | 6.85 | 21 | 21 | 49.47 | Unstable | 65.73 | −0.85 |
| AtSUMO5 | C_524_H_844_N_148_O_164_S_8_ | 12.10 | 8.91 | 12 | 15 | 51.41 | Unstable | 64.07 | −0.61 |
| AtSUMO6 | C_588_H_934_N_170_O_175_S_7_ | 13.41 | 9.13 | 18 | 21 | 43.92 | Unstable | 62.28 | −0.76 |
| AtSUMO7 | C_478_H_756_N_136_O_145_S_6_ | 10.92 | 6.03 | 15 | 13 | 30.69 | Stable | 76.00 | −0.55 |
| AtSUMO8 | C_498_H_782_N_138_O_150_S_5_ | 11.26 | 6.05 | 16 | 14 | 53.29 | Unstable | 76.29 | −0.56 |
