# Supplementary material for: Identification and characterization of novel SUMO genes in bread wheat
Source: PeerJ. 2025 Nov 28;13:e20432. doi: 10.7717/peerj.20432 (PMC12667693; doi:10.7717/peerj.20432)
Supplement: Supplemental Information 3 — AC Atomic composition; C* Carbon; H* Hydrogen; N* Nitrogen; O* Oxygen; S* Sulfur; TNA Total number of atoms; AAC Amino acid composition; A Alanine; R Arginine; N Asparagin; D Aspartic; C Cysteine ; Q Glutamin; E Glutamic; G Glycine; H Histidine; I Isoleucine; L Leucine; K Lysine; M Methionine; F Phenylalanine; P Proline; S Serine; T Threonine; W Tryptophan; Y Tyrosin; V Valine; NAA Number of amino acids [file peerj-13-20432-s003.docx]

| **SUMOs** | **AC** | | | | | **TNA** | **AAC** | | | | | | | | | | | | | | | | | | | | **NAA** |
| --- | --- | --- | --- | --- | --- | --- | --- | --- | --- | --- | --- | --- | --- | --- | --- | --- | --- | --- | --- | --- | --- | --- | --- | --- | --- | --- | --- |
|  | **C*** | **H*** | **N*** | **O*** | **S*** |  | **A** | **R** | **N** | **D** | **C** | **Q** | **E** | **G** | **H** | **I** | **L** | **K** | **M** | **F** | **P** | **S** | **T** | **W** | **Y** | **V** |  |
| TaSUMO1 | 479 | 764 | 142 | 156 | 6 | 1547 | 6 | 7 | 4 | 10 | 2 | 5 | 8 | 12 | 2 | 4 | 8 | 7 | 4 | 5 | 4 | 4 | 4 | 0 | 1 | 4 | 101 |
| TaSUMO2 | 489 | 788 | 146 | 160 | 6 | 1589 | 9 | 7 | 3 | 9 | 1 | 5 | 9 | 13 | 2 | 4 | 8 | 8 | 5 | 4 | 5 | 5 | 4 | 0 | 1 | 3 | 105 |
| TaSUMO3 | 599 | 964 | 176 | 203 | 4 | 1946 | 15 | 6 | 4 | 8 | 1 | 4 | 13 | 11 | 5 | 7 | 6 | 9 | 3 | 3 | 5 | 13 | 6 | 0 | 2 | 10 | 131 |
| TaSUMO4 | 512 | 809 | 139 | 151 | 8 | 1619 | 7 | 6 | 0 | 10 | 1 | 2 | 5 | 10 | 3 | 2 | 8 | 7 | 7 | 5 | 7 | 6 | 5 | 1 | 2 | 11 | 105 |
| TaSUMO5 | 524 | 832 | 142 | 150 | 10 | 1658 | 3 | 5 | 2 | 8 | 0 | 2 | 5 | 12 | 4 | 2 | 7 | 8 | 10 | 4 | 6 | 4 | 6 | 1 | 3 | 14 | 106 |
| TaSUMO6 | 576 | 903 | 157 | 180 | 6 | 1822 | 10 | 6 | 1 | 8 | 0 | 3 | 13 | 7 | 4 | 2 | 7 | 7 | 6 | 4 | 6 | 8 | 7 | 3 | 1 | 14 | 117 |
| TaSUMO7 | 591 | 952 | 184 | 180 | 5 | 1912 | 6 | 13 | 1 | 13 | 0 | 3 | 6 | 16 | 5 | 3 | 13 | 6 | 5 | 4 | 2 | 9 | 4 | 2 | 1 | 11 | 123 |
| OsSUMO1 | 464 | 744 | 138 | 153 | 7 | 1506 | 9 | 6 | 4 | 9 | 2 | 5 | 9 | 13 | 2 | 4 | 8 | 7 | 5 | 4 | 3 | 3 | 3 | 0 | 1 | 3 | 100 |
| OsSUMO2 | 471 | 756 | 138 | 156 | 5 | 1526 | 8 | 5 | 4 | 9 | 1 | 6 | 8 | 12 | 2 | 5 | 8 | 8 | 4 | 4 | 4 | 6 | 3 | 0 | 1 | 3 | 101 |
| OsSUMO3 | 542 | 834 | 156 | 173 | 4 | 1709 | 10 | 11 | 0 | 11 | 0 | 5 | 10 | 14 | 1 | 2 | 7 | 4 | 4 | 5 | 2 | 2 | 8 | 2 | 5 | 7 | 110 |
| OsSUMO4 | 532 | 859 | 145 | 176 | 5 | 1717 | 10 | 5 | 1 | 11 | 3 | 6 | 6 | 9 | 1 | 7 | 8 | 7 | 2 | 4 | 6 | 7 | 11 | 0 | 2 | 8 | 114 |
| OsSUMO5 | 545 | 858 | 142 | 164 | 10 | 1719 | 5 | 6 | 1 | 11 | 1 | 3 | 5 | 10 | 2 | 6 | 7 | 6 | 9 | 7 | 6 | 4 | 10 | 0 | 3 | 8 | 110 |
| OsSUMO6 | 657 | 995 | 183 | 195 | 4 | 2034 | 11 | 11 | 2 | 7 | 0 | 5 | 12 | 16 | 1 | 3 | 9 | 5 | 4 | 5 | 6 | 5 | 9 | 6 | 5 | 8 | 130 |
| OsSUMO7 | 492 | 795 | 127 | 146 | 5 | 1565 | 2 | 2 | 3 | 8 | 2 | 1 | 9 | 12 | 3 | 3 | 17 | 9 | 3 | 3 | 3 | 4 | 3 | 2 | 0 | 11 | 100 |
| AtSUMO1 | 462 | 740 | 140 | 158 | 6 | 1506 | 8 | 6 | 5 | 11 | 1 | 6 | 7 | 12 | 2 | 4 | 7 | 7 | 5 | 4 | 2 | 5 | 4 | 0 | 1 | 3 | 100 |
| AtSUMO2 | 578 | 903 | 159 | 177 | 6 | 1823 | 8 | 6 | 5 | 11 | 2 | 6 | 8 | 10 | 2 | 5 | 10 | 9 | 4 | 9 | 3 | 4 | 6 | 1 | 1 | 6 | 116 |
| AtSUMO3 | 550 | 866 | 154 | 170 | 7 | 1747 | 8 | 6 | 5 | 13 | 2 | 6 | 6 | 9 | 2 | 5 | 11 | 9 | 5 | 5 | 4 | 5 | 1 | 1 | 3 | 5 | 111 |
| AtSUMO4 | 586 | 939 | 173 | 185 | 5 | 1888 | 4 | 11 | 3 | 9 | 0 | 3 | 12 | 9 | 3 | 4 | 8 | 10 | 5 | 5 | 2 | 10 | 6 | 1 | 3 | 9 | 117 |
| AtSUMO5 | 524 | 844 | 148 | 164 | 8 | 1688 | 6 | 5 | 3 | 4 | 2 | 4 | 8 | 7 | 4 | 4 | 7 | 10 | 6 | 2 | 5 | 11 | 8 | 0 | 5 | 7 | 108 |
| AtSUMO6 | 588 | 934 | 170 | 175 | 7 | 1874 | 2 | 11 | 3 | 8 | 1 | 4 | 10 | 8 | 2 | 4 | 7 | 10 | 6 | 7 | 3 | 8 | 7 | 2 | 2 | 9 | 114 |
| AtSUMO7 | 478 | 756 | 136 | 145 | 6 | 1521 | 6 | 6 | 2 | 11 | 2 | 6 | 4 | 4 | 4 | 10 | 4 | 7 | 4 | 5 | 5 | 6 | 3 | 0 | 2 | 4 | 95 |
| AtSUMO8 | 498 | 782 | 138 | 150 | 5 | 1573 | 2 | 6 | 4 | 8 | 1 | 3 | 8 | 6 | 4 | 6 | 8 | 8 | 4 | 7 | 3 | 8 | 3 | 0 | 2 | 6 | 97 |
