# Supplementary material for: Identification and characterization of novel SUMO genes in bread wheat
Source: PeerJ. 2025 Nov 28;13:e20432. doi: 10.7717/peerj.20432 (PMC12667693; doi:10.7717/peerj.20432)
Supplement: Supplemental Information 4 — A Alanine; R Arginine; N Asparagin; D Aspartic; C Cysteine; Q Glutamin; E Glutamic; G Glycine; H Histidine; I Isoleucine; L Leucine; K Lysine; M Methionine; F Phenylalanine; P Proline; S Serine; T Threonine; W Tryptophan; Y Tyrosin; V Valin [file peerj-13-20432-s004.docx]

|  | **A** | **R** | **N** | **D** | **C** | **Q** | **E** | **G** | **H** | **I** | **L** | **K** | **M** | **F** | **P** | **S** | **T** | **W** | **Y** | **V** |
| --- | --- | --- | --- | --- | --- | --- | --- | --- | --- | --- | --- | --- | --- | --- | --- | --- | --- | --- | --- | --- |
| TaSUMO1 | 5.90% | 6.90% | 4.00% | 9.90% | 2.00% | 5.00% | 7.90% | 11.90% | 2.00% | 4.00% | 7.90% | 6.90% | 4.00% | 5.00% | 4.00% | 4.00% | 4.00% | 0.00% | 1.00% | 4.00% |
| TaSUMO2 | 8.60% | 6.70% | 2.90% | 8.60% | 1.00% | 4.80% | 8.60% | 12.40% | 1.90% | 3.80% | 7.60% | 7.60% | 4.80% | 3.80% | 4.80% | 4.80% | 3.80% | 0.00% | 1.00% | 2.90% |
| TaSUMO3 | 11.50% | 4.60% | 3.10% | 6.10% | 0.80% | 3.10% | 9.90% | 8.40% | 3.80% | 5.30% | 4.60% | 6.90% | 2.30% | 2.30% | 3.80% | 9.90% | 4.60% | 0.00% | 1.50% | 7.60% |
| TaSUMO4 | 6.70% | 5.70% | 0.00% | 9.50% | 1.00% | 1.90% | 4.80% | 9.50% | 2.90% | 1.90% | 7.60% | 6.70% | 6.70% | 4.80% | 6.70% | 5.70% | 4.80% | 1.00% | 1.90% | 10.50% |
| TaSUMO5 | 2.80% | 4.70% | 1.90% | 7.50% | 0.00% | 1.90% | 4.70% | 11.30% | 3.80% | 1.90% | 6.60% | 7.50% | 9.40% | 3.80% | 5.70% | 3.80% | 5.70% | 0.90% | 2.80% | 13.20% |
| TaSUMO6 | 8.50% | 5.10% | 0.90% | 6.80% | 0.00% | 2.60% | 11.10% | 6.00% | 3.40% | 1.70% | 6.00% | 6.00% | 5.10% | 3.40% | 5.10% | 6.80% | 6.00% | 2.60% | 0.90% | 12.00% |
| TaSUMO7 | 4.90% | 10.60% | 0.80% | 10.60% | 0.00% | 2.40% | 4.90% | 13.00% | 4.10% | 2.40% | 10.60% | 4.90% | 4.10% | 3.30% | 1.60% | 7.30% | 3.30% | 1.60% | 0.80% | 8.90% |
| OsSUMO1 | 9.00% | 6.00% | 4.00% | 9.00% | 2.00% | 5.00% | 9.00% | 13.00% | 2.00% | 4.00% | 8.00% | 7.00% | 5.00% | 4.00% | 3.00% | 3.00% | 3.00% | 0.00% | 1.00% | 3.00% |
| OsSUMO2 | 7.90% | 5.00% | 4.00% | 8.90% | 1.00% | 5.90% | 7.90% | 11.90% | 2.00% | 5.00% | 7.90% | 7.90% | 4.00% | 4.00% | 4.00% | 5.90% | 3.00% | 0.00% | 1.00% | 3.00% |
| OsSUMO3 | 9.10% | 10.00% | 0.00% | 10.00% | 0.00% | 4.50% | 9.10% | 12.70% | 0.90% | 1.80% | 6.40% | 3.60% | 3.60% | 4.50% | 1.80% | 1.80% | 7.30% | 1.80% | 4.50% | 6.40% |
| OsSUMO4 | 8.80% | 4.40% | 0.90% | 9.60% | 2.60% | 5.30% | 5.30% | 7.90% | 0.90% | 6.10% | 7.00% | 6.10% | 1.80% | 3.50% | 5.30% | 6.10% | 9.60% | 0.00% | 1.80% | 7.00% |
| OsSUMO5 | 4.50% | 5.50% | 0.90% | 10.00% | 0.90% | 2.70% | 4.50% | 9.10% | 1.80% | 5.50% | 6.40% | 5.50% | 8.20% | 6.40% | 5.50% | 3.60% | 9.10% | 0.00% | 2.70% | 7.30% |
| OsSUMO6 | 8.50% | 8.50% | 1.50% | 5.40% | 0.00% | 3.80% | 9.20% | 12.30% | 0.80% | 2.30% | 6.90% | 3.80% | 3.10% | 3.80% | 4.60% | 3.80% | 6.90% | 4.60% | 3.80% | 6.20% |
| OsSUMO7 | 2.00% | 2.00% | 3.00% | 8.00% | 2.00% | 1.00% | 9.00% | 12.00% | 3.00% | 3.00% | 17.00% | 9.00% | 3.00% | 3.00% | 3.00% | 4.00% | 3.00% | 2.00% | 0.00% | 11.00% |
| AtSUMO1 | 8.00% | 6.00% | 5.00% | 11.00% | 1.00% | 6.00% | 7.00% | 12.00% | 2.00% | 4.00% | 7.00% | 7.00% | 5.00% | 4.00% | 2.00% | 5.00% | 4.00% | 0.00% | 1.00% | 3.00% |
| AtSUMO2 | 6.90% | 5.20% | 4.30% | 9.50% | 1.70% | 5.20% | 6.90% | 8.60% | 1.70% | 4.30% | 8.60% | 7.80% | 3.40% | 7.80% | 2.60% | 3.40% | 5.20% | 0.90% | 0.90% | 5.20% |
| AtSUMO3 | 7.20% | 5.40% | 4.50% | 11.70% | 1.80% | 5.40% | 5.40% | 8.10% | 1.80% | 4.50% | 9.90% | 8.10% | 4.50% | 4.50% | 3.60% | 4.50% | 0.90% | 0.90% | 2.70% | 4.50% |
| AtSUMO4 | 3.40% | 9.40% | 2.60% | 7.70% | 0.00% | 2.60% | 10.30% | 7.70% | 2.60% | 3.40% | 6.80% | 8.50% | 4.30% | 4.30% | 1.70% | 8.50% | 5.10% | 0.90% | 2.60% | 7.70% |
| AtSUMO5 | 5.60% | 4.60% | 2.80% | 3.70% | 1.90% | 3.70% | 7.40% | 6.50% | 3.70% | 3.70% | 6.50% | 9.30% | 5.60% | 1.90% | 4.60% | 10.20% | 7.40% | 0.00% | 4.60% | 6.50% |
| AtSUMO6 | 1.80% | 9.60% | 2.60% | 7.00% | 0.90% | 3.50% | 8.80% | 7.00% | 1.80% | 3.50% | 6.10% | 8.80% | 5.30% | 6.10% | 2.60% | 7.00% | 6.10% | 1.80% | 1.80% | 7.90% |
| AtSUMO7 | 6.30% | 6.30% | 2.10% | 11.60% | 2.10% | 6.30% | 4.20% | 4.20% | 4.20% | 10.50% | 4.20% | 7.40% | 4.20% | 5.30% | 5.30% | 6.30% | 3.20% | 0.00% | 2.10% | 4.20% |
| AtSUMO8 | 2.10% | 6.20% | 4.10% | 8.20% | 1.00% | 3.10% | 8.20% | 6.20% | 4.10% | 6.20% | 8.20% | 8.20% | 4.10% | 7.20% | 3.10% | 8.20% | 3.10% | 0.00% | 2.10% | 6.20% |
