# Supplementary figures and images for: Identification and characterization of novel SUMO genes in bread wheat
Source: PeerJ. 2025 Nov 28;13:e20432. doi: 10.7717/peerj.20432 (PMC12667693; doi:10.7717/peerj.20432)

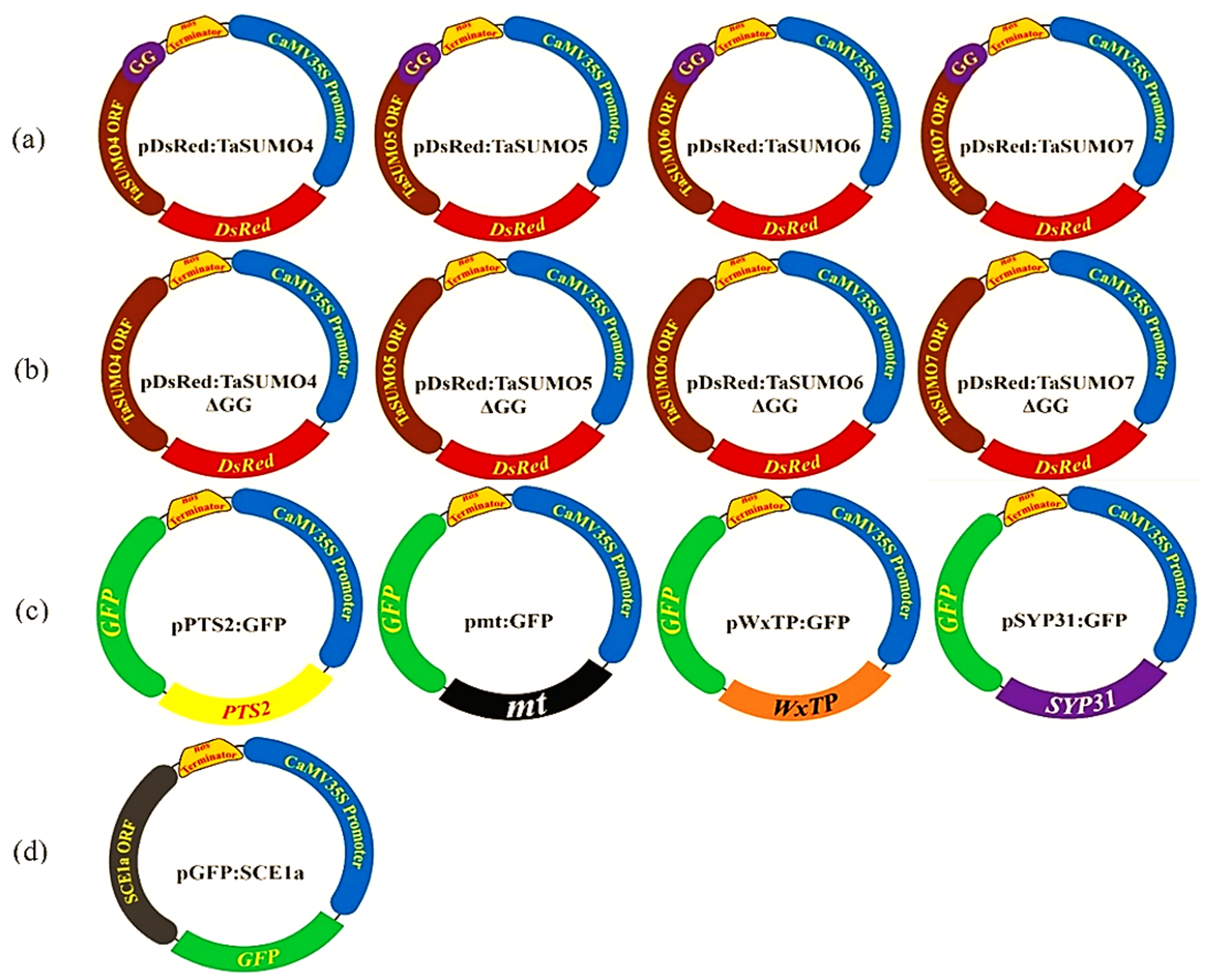

Supplement: Supplemental Information 5 — (a) Construction of expression plasmid harboring TaSUMO4, 5, 6, 7 gene s. (b) Construction of expression plasmid harboring TaSUMO4, 5, 6, 7 gene with GG Deletion. (c) Construction of expression plasmid PTS2:GFP, mt:GFP, WxTP:GFP, SYP31:GFP. (d) Construction of expression plasmid harboring SCE1a gene [file peerj-13-20432-s005.png]
